# Epidemiology of COVID-19 in the Kingdom of Saudi Arabia: An Ecological Study

**DOI:** 10.3389/fpubh.2020.00506

**Published:** 2020-09-17

**Authors:** Mohammad H. Alyami, Abdallah Y. Naser, Mohamed A. A. Orabi, Hassan Alwafi, Hamad S. Alyami

**Affiliations:** ^1^Department of Pharmaceutics, College of Pharmacy, Najran University, Najran, Saudi Arabia; ^2^Faculty of Pharmacy, Isra University, Amman, Jordan; ^3^Department of Pharmacognosy, College of Pharmacy, Najran University, Najran, Saudi Arabia; ^4^Department of Pharmacognosy, Faculty of Pharmacy, Al-Azhar University, Asyut, Egypt; ^5^Department of Pharmacology and Toxicology, College of Medicine, Umm Alqura University, Mecca, Saudi Arabia

**Keywords:** COVID-19, epidemiology, pandemic, Saudi Arabia, trend

## Abstract

**Objectives:** Considering the transmissible nature of COVID-19 it is important to explore the trend of the epidemiology of the disease in each country and act accordingly. This study aimed to examine the trend of COVID-19 epidemiology in the Kingdom of Saudi Arabia in term of its incidence rate, recovery rate, and mortality rate.

**Material and Methods:** We conducted an observational study using publicly available national data taken from the Saudi Ministry of Health for the period between 3 March and 7 June 2020. The number of newly confirmed cases, active cases, critical cases, percentage of cases stratified by age group [adults, children, and elderly] and gender were extracted from the reports of the Saudi Ministry of Health.

**Results:** During the study period, the total number of confirmed cases with COVID-19 rose from one on 2 March 2020 to 101,914 on 7 June, representing an average of 1,039 new cases per day, [trend test, *p* < 0.000]. Despite the increase in the number of newly confirmed daily cases of COVID-19, the number of reported daily active cases started to stabilize after 2 months from the start of the pandemic in the country and the overall recovery rate was 71.4%. The mortality rate decreased by 6.4% during the study period. COVID-19 was more common among adults and males compared to other demographic groups.

**Conclusion:** The epidemiological status of COVID-19 in the Kingdom of Saudi Arabia showing promising improvement. Males and adults accounted for the majority of COVID-19 cases in the KSA. Further studies are recommended to be conducted at the patient level to identify other patient groups who are at higher risk of getting infected with COVID-19, and for whom the best pharmacological intervention could be provided.

## Introduction

The novel coronavirus disease 2019 (COVID-19) was first isolated from biological samples in Wuhan, China, in December 2019. The virus was identified as a member of the genus betacoronavirus, grouping it with Severe Acute Respiratory Syndrome (SARS) and Middle East Respiratory Syndrome (MERS) (1). The virus spread internationally within 1 month of first being identified, being transmitted via close human-to-human contact (2). The World Health Organization (WHO) declared COVID-19 (SARS-CoV-2) a Public Health Emergency of International Concern on 1 February 2020. Over 200 countries have confirmed cases to date, including countries from Asia, Europe, North America and the Middle East.

The ongoing explosive spread of COVID-19 and the new hotspots beyond the first city of Wuhan, especially in the United States (US), Russia, United Kingdom (UK), Italy, Spain, Brazil, and its introduction to the Middle Eastern countries calls for additional regional actions to stem its further spread (3). For the first time in the eight decades of the Muslim pilgrimage to the holy sites in the Kingdom of Saudi Arabia (KSA), on 27 February 2020, KSA placed restrictions on inbound Umrah pilgrimage, placed a ban on inbound travel of persons coming from COVID-19-affected countries and restrictions on travel of Gulf Cooperation Council (GCC) citizens who have traveled to COVID-19-affected countries. By 7 June 2020, the number of persons infected by the virus in the KSA has reached more than 100,000 and Saudi authorities have reported 283 deaths from the virus, most of which were in the main cities including Mecca, Riyadh, Jeddah, and Medina (4).

The global outbreak of COVID-19 has been a matter of international concern as the disease is spreading very fast. Considering the transmissible nature of the disease, which has had a massive impact worldwide, there is a crucial need to explore the trend of the epidemiology of the disease in the KSA. This will help clinicians to establish risk stratification of COVID-19 patients as early as possible, calling on the community to pay more attention to defending the more susceptible groups from the virus in order to decrease its prevalence. Due to differences in the physiological structure of women and men, gender differences play an indisputable role in the pandemic of the disease (5). Also, aging is connected with a number of variations in pulmonary physiology, pathology and function, throughout the period of lung infection. The age-related alterations in sensitivity and tolerance may lead to an increased rate of death in aged people (6).

To the best of our knowledge, there is no previous study that has investigated the characteristics of the epidemiology of COVID-19 in the KSA. This study aimed to examine the trend of COVID-19 epidemiology in the KSA in term of its incidence rate, recovery rate, and mortality rate. In addition, this study aimed to explore the gender and age differences in term of the epidemiology of the disease, and the trend of COVID-19 mortality.

## Materials and Methods

### Study Sources and the Population

This was a secular trend study using publicly available national data taken from the Saudi Ministry of Health for the period between 2 March and 7 June 2020 (7, 8). The Saudi Ministry of Health provided detailed data on the incidence of COVID-19 in the Kingdom daily, with the following details: (a) the number of newly confirmed cases, (b) number of active cases, (c) number of critical cases, (d) percentage of cases stratified by age group [adults, children, and elderly] (available from 2 May until 25 May), and (e) percentage of cases stratified by gender (available from 2 May until 25 May). In addition, the number of newly confirmed cases stratified by city is available starting from 25 March onwards. The Saudi Ministry of Health report on COVID-19 cases is based on real-time (RT)-PCR obtained through nasopharyngeal swabs, which were processed and validated through a regional lab. Confirmed COVID-19 case is defined as a case with positive real-time (RT)-PCR sample obtained through nasopharyngeal swabs. Active case is defined as a COVID-19 case that is still under medical supervision without two negative real-time (RT)-PCR samples. Critical case is defined as a COVID-19 case which required intensive care unit (ICU) admission.

### Statistical Analysis

The trend of the epidemiology of COVID-19 was presented graphically, showing the number of daily confirmed cases during a period of 98 days (between 2 March and 7 June 2020). The same procedure was followed to present the total number of confirmed cases and number of active cases. COVID-19 recovery rates, with 95% confidence intervals (CIs), were calculated using the number of daily recovered cases divided by the total number of active cases in the same day. The same procedure was followed to calculate the mortality rate and the rate of critical cases daily. The chi-squared test was used to assess the difference between the recovery rates on 2 March and 7 June 2020. Similarly, the chi-squared test was used to assess the difference in the mortality rate and the rate of critical cases during the study period. The trends in the epidemiology of COVID-19 were assessed using a Poisson model. SPSS (Statistical Package for the Social Sciences) version 25.0 software was used to perform all statistical analysis.

## Results

**Figure 1:**
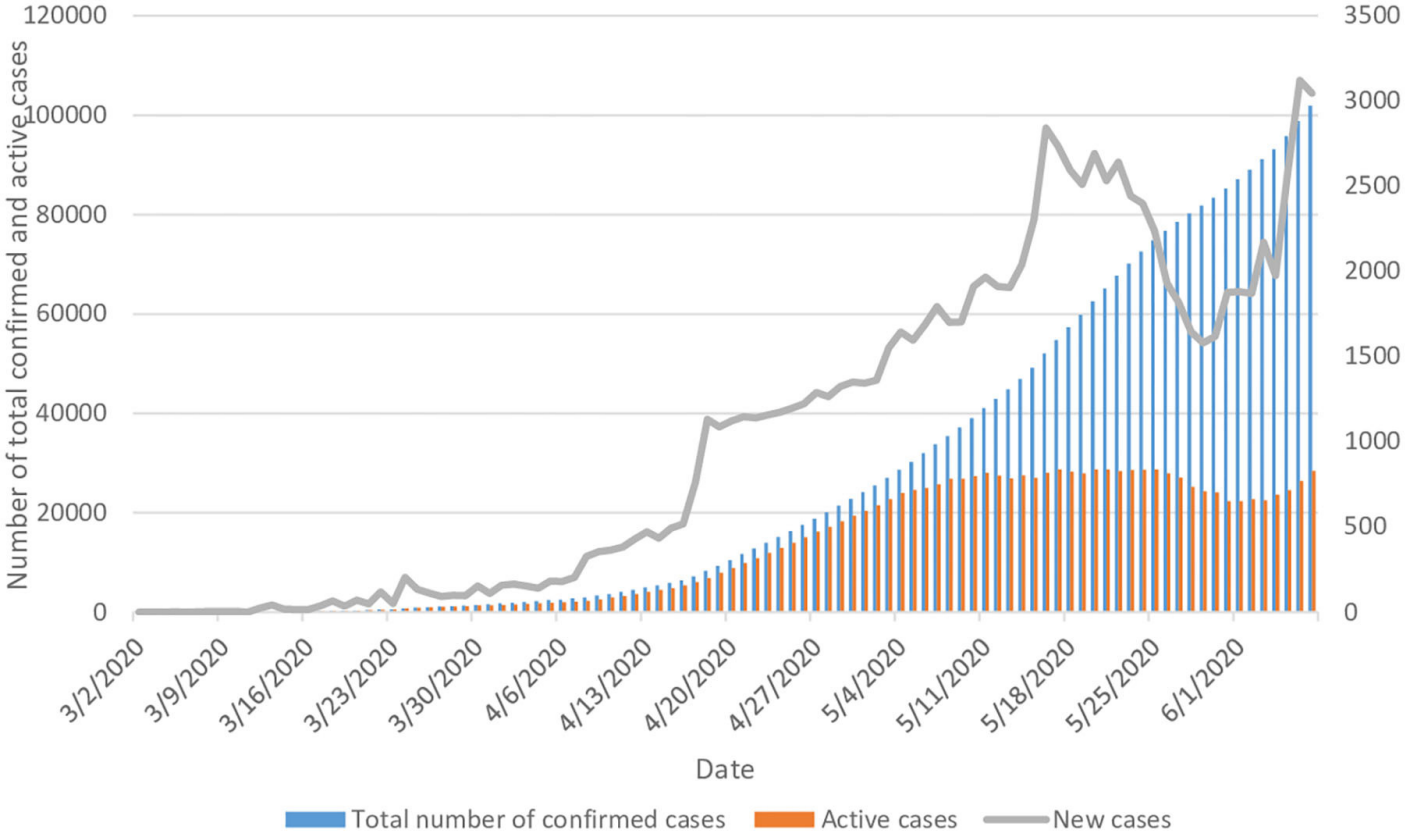
Trends of the epidemiology of COVID-19 in Saudi Arabia.

The total number of confirmed cases with COVID-19 rose from one on 2 March 2020 to 101,914 on 7 June, representing an average of 1,039 new cases per day, [trend test, *p* < 0.000]. Compared to 2 March (the date of the first reported COVID-19 case) the number of new daily cases on 7 June had reached 3,045 cases. The number of daily active cases showed a continuous increase for a duration of 76 days until 16 May, then it started decreasing until 29 May; from this date the number of daily active cases started to increase again to peak on 6 June (Figure 1).

**Figure 2:**
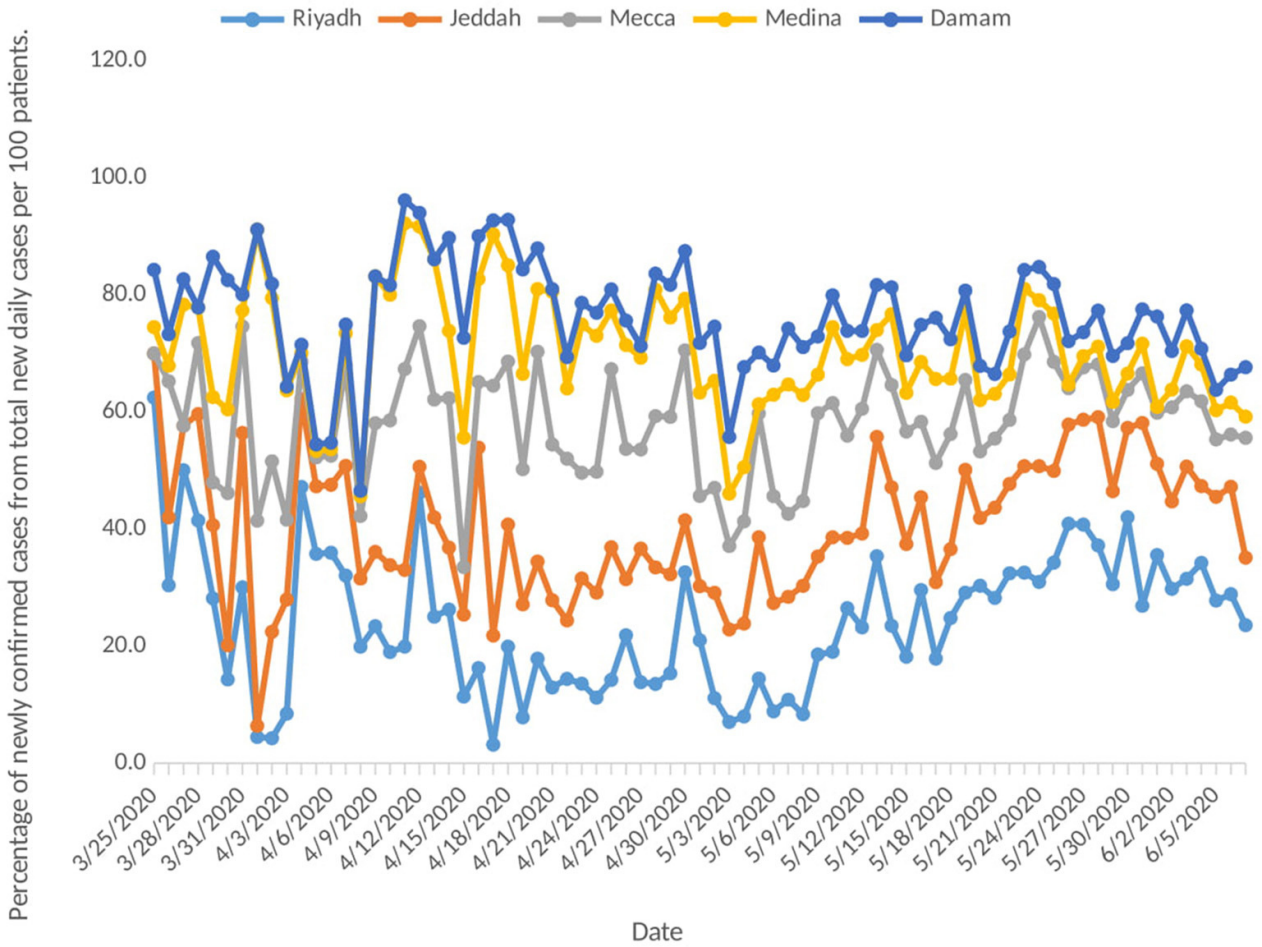
Incidence rate of COVID-19 stratified by city (five main cities in Saudi Arabia).

Despite the increase in the number of newly confirmed daily cases of COVID-19, the number of reported daily active cases started to stabilize from 8 May onwards, fluctuating between 26,856 and 28,358 on 7 June (Figure 1). As we can see in Figure 2, the epidemiological patterns of COVID-19 in the five main cities in the KSA (Riyadh, Jeddah, Mecca, Medina, and Damam) were not similar throughout the study period. The highest percentage of new daily cases was in Riyadh (average 24.2%), followed by Mecca (17.7%) and Jeddah (16.2%). In addition, Medina contributed an average of 12.1%, and Damam 6.1% of the daily new cases.

**Figure 3:**
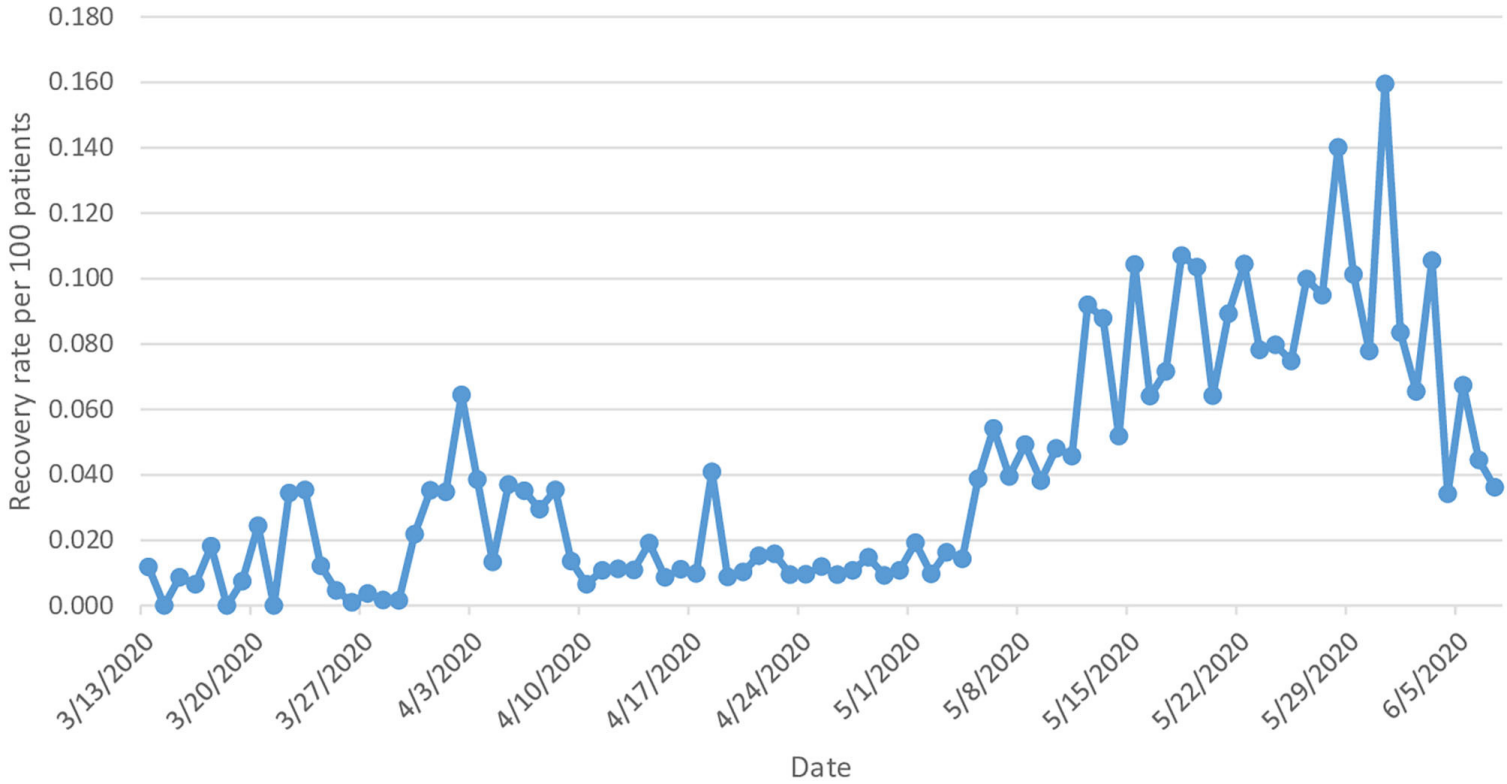
Recovery rate from COVID-19.

During the study period, a total of 72,817 patients have recovered from COVID-19 as of 7 June, out of a total of 101,914 confirmed cases, representing a recovery rate of 71.4%. The recovery rate during the study period increased 2-fold from 1.18 (95% CI 0.93–1.43) on 13 March [one recovered case] to 3.61 times (95% CI 3.57–3.66) on 7 June [1,026 recovered cases] (Figure 3).

**Figure 4:**
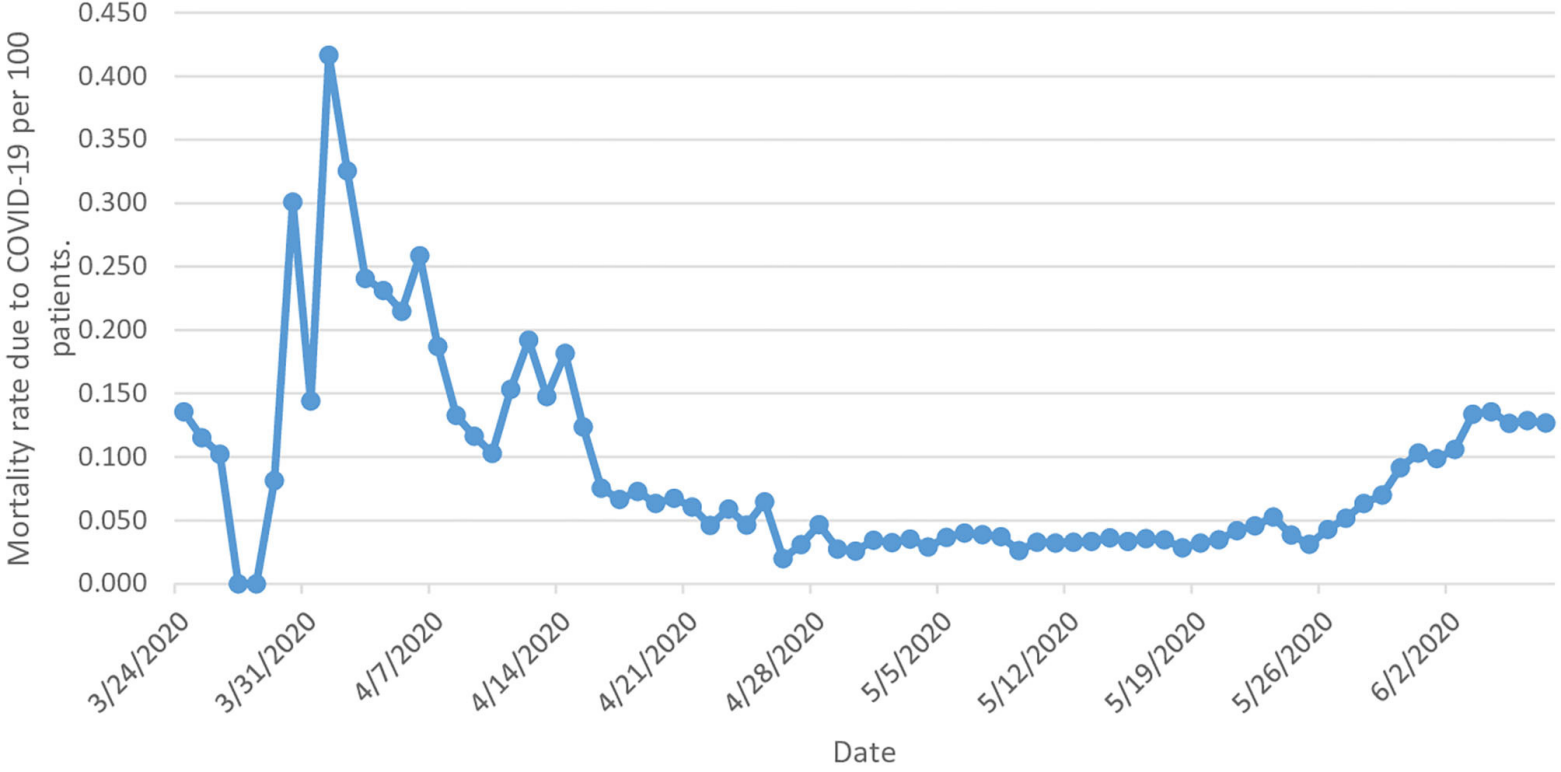
Mortality rates due to COVID-19.

Mortality rates due to COVID-19 have been fluctuating, starting from 2 March, reaching the peak rate of deaths on 1 April (0.417 per 100 patients), which was followed by nonlinear reduction until 26 April (0.020 per 100 patients). Starting from 29 April, the mortality rate remained relatively constant, fluctuating between 0.027 per 100 patients and 0.028 per 100 patients on 18 May. This was followed by another increase in the mortality rate, which reached 0.127 per 100 patients on 7 June. Despite that, the number of daily deaths showed a constant increase during the study period, starting from one death reported on 24 March to 36 daily deaths by 7 June (712 deaths in total), with an average of seven deaths per day. The mortality rate decreased by 6.4% from 0.136 (95% CI 0.118–0.153) per 100 patients [with one death] to 0.127 (95% CI 0.124–0.130) per 100 patients between 24 March and 7 June [with 36 deaths] (Figure 4).

**Figure 5:**
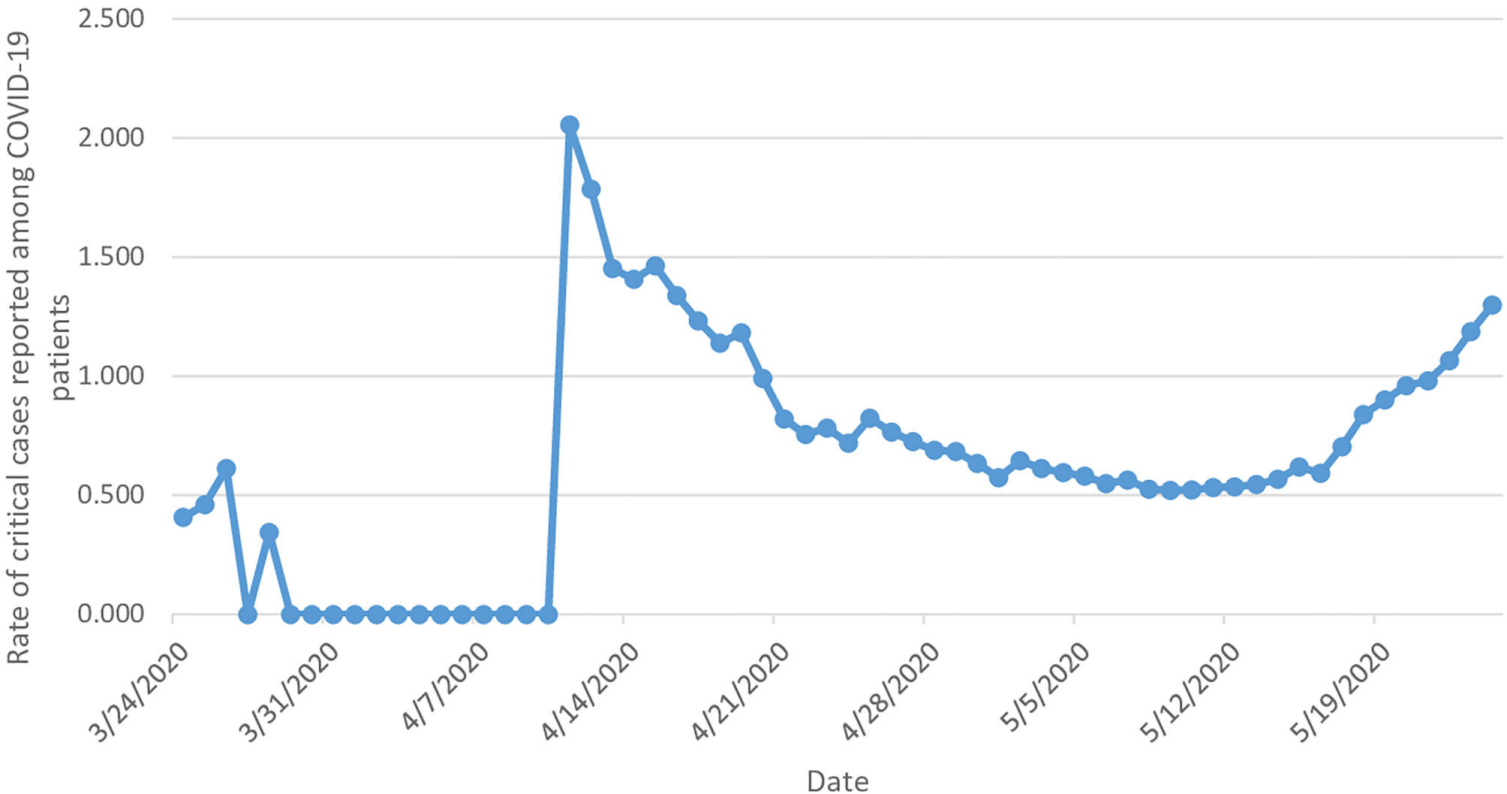
Rate of daily reported critical cases among COVID-19 patients.

The rate of critical cases from the date of reporting the first critical COVID-19 case (24 March) until the end of the study period increased by 1.3 times, from 0.407 (95% CI 0.381–0.432) [with three critical cases] to 1.337 (95% CI 1.242–1.432) [with 384 critical cases] (Figure 5).

**Figure 6:**
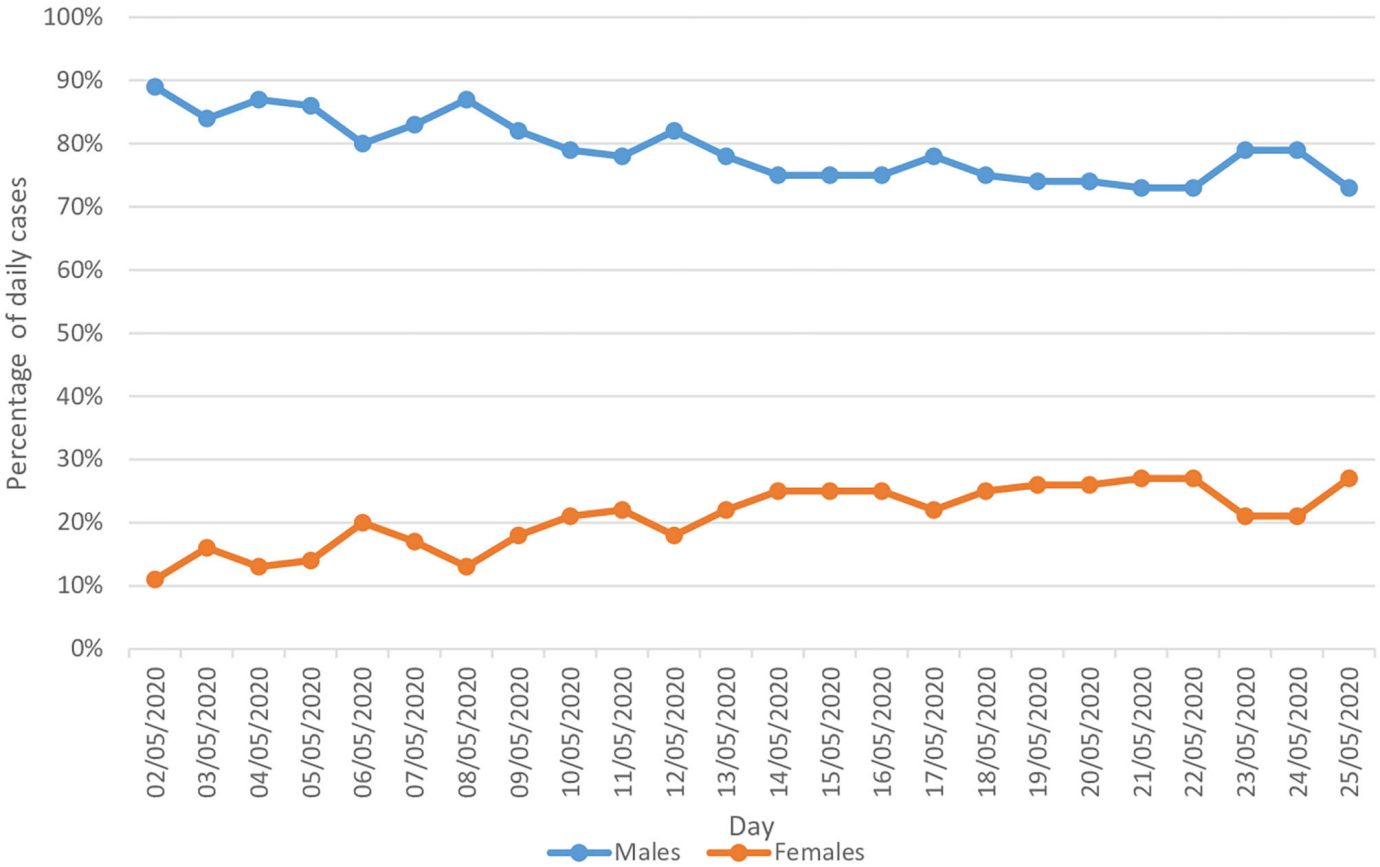
Rate of the epidemiology of COVID-19 daily cases stratified by gender.

During the 24 days of the study period (between 2 May and 25 May, the period for which the data were available stratified by gender), COVID-19 was clearly more prevalent among males compared to females. On average, males contributed to 79.0% of the cases, compared to 21.0% for females. The percentage of males in the total reported daily cases decreased by 18.0% (from 89.0% on 2 May to 73.0% on 25 May). On the other hand, the percentage of females in the total reported daily cases increased by 1.5 times, from 11.0 to 27.0% (Figure 6).

**Figure 7:**
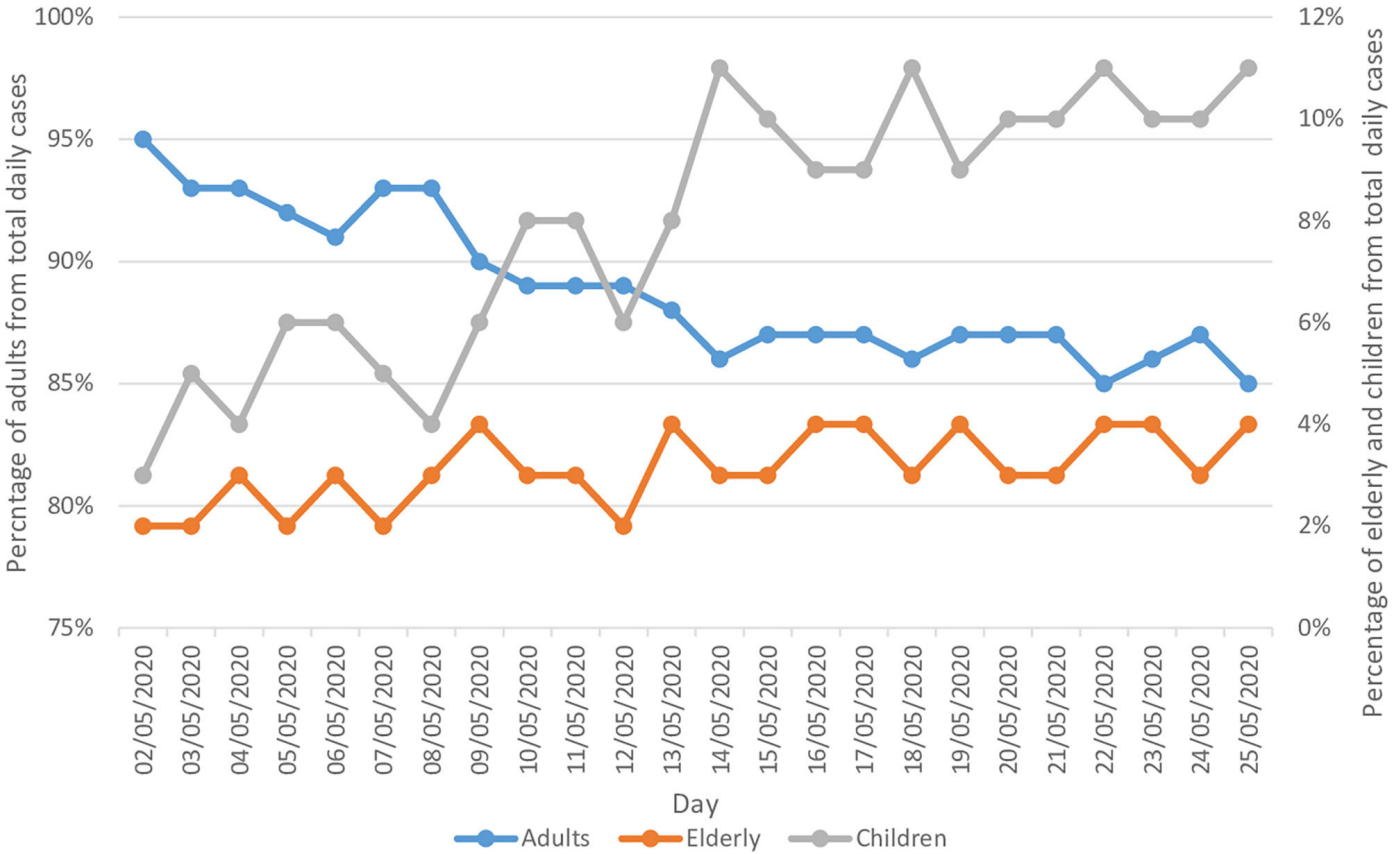
Rate of the epidemiology of COVID-19 daily cases stratified by age group.

During the same period of the study, COVID-19 was clearly more prevalent among adults compared to children and the elderly population. On average adults contributed to 89.0% of the daily reported cases, followed by children and the elderly, with 8.0 and 3.0%, respectively. The percentage of adults in the total reported daily cases decreased by 10.5% (from 95.0% on 2 May to 85.0% on 25 May). On the other hand, the percentage of children and the elderly in the total reported daily cases doubled and trebled, respectively (Figure 7).

## Discussion

Our study explored the trend of COVID-19 epidemiology in the KSA in term of its incidence rate, recovery rate, and mortality rate. The key findings were: (1) the epidemiological status in KSA reached a steady level after 2 months from the beginning of the pandemic, due to the implementation of successful healthcare and treatment protocols, (2) the international travel restrictions and household quarantine were effective ways to control the epidemic of COVID-19 the KSA, (3) the rates of critical cases and mortality in KSA are at a low level, due to the younger population in Saudi Arabia compared to European and Asian countries, and the effective control measures taken by the government, and (4) in the KSA, COVID-19 was more common among adults and males compared to other demographic groups.

Despite the high number of preventive and control measures that have been taken by the Saudi Arabian government, the results of this study demonstrate an exponential increase in the total number of newly confirmed cases. The results confirm the rapid spread of the disease among the citizens in the KSA during the first 2 months of the pandemic (until 8 May). The rate of active COVID-19 cases showed an exponential increase during the first 2 months, which started to stabilize and increase less sharply from 8 May onwards, probably due to the continuous increase in the recovery rate from the disease. This could be explained by advances in the Saudi healthcare system, which provides advanced medical care for the patients that decreases the probability of life-threatening complications, or increases in citizens' adherence to personal protective measures. Despite applying the same restrictions and preventive measures across all the cities in the KSA, the incidence of COVID-19 was not similar across different cities. This may be due to differences in citizens' adherence to government restrictions. In addition, there are many differences between these five main cities in term of population density and diversity in the nationality of their inhabitants. Diversity in the culture between the individuals lead to different implications on the epidemiology of diseases as it affects their attitudes, knowledge and practices toward the disease (9–12).

The current study suggests potential risk factors among COVID-19 infected cases. Adults contributed to the highest proportion (on average 89.0%) of the daily reported new COVID-19 cases, compared to children and elderly. Therefore, they are considered the most vulnerable individuals to get infected with COVID-19. This was confirmed by earlier studies which showed that mortality of COVID-19 is linked with age: 80% of reported deaths in China were of individuals aged over 65 years old, and up to 15% of the deaths in the US were among adults over 70 years (13, 14). Similarly, in another large database study that included data from 17 million patients in the UK, the authors of the study highlighted that patients aged >60 years were at higher risk of hospital mortality due to COVID-19, specifically patients aged >80 HR 12.64 (95% CI 11.19–14.28) (15). Several recent studies have speculated on the reasons for age being a risk factor of COVID-19. For instance, the response of the immune system in adults may undergo several changes over the years, including the production of T and B lymphocytes, and the coordination of the immune system (16), which may lead to excessive immune response and further complications such as hypercoagulability and endotheliopathy (17). Chronic illnesses have been linked with poorer outcome in patients with COVID-19, and comorbidities are more common among the elderly compared to younger populations (15, 18, 19). Besides this, older populations tend to have a higher risk of mortality associated with influenza and other respiratory viruses which are similar to documented in SARS-COV2 (20). However, confirmed cases within the child population are usually less severe than for adults (14, 21). More than 90% of infected children are asymptomatic or have mild to moderate disease (21). COVID-19 cases for infants are few, with mild illness (22). Similar findings have stated that SARS-CoV-2 preferentially infects older adult males, with rare cases reported in children (3, 6, 23). These are all in line with our study findings.

Another suggested risk factor that emerged from our study is that there is a gender difference in term of COVID-19 epidemiology, with males more susceptible to COVID-19 infection than females. Our study found that the highest proportion of COVID-19 cases were among the male population (on average 79% of the cases) compared to only 21% for females. Various epidemiological and population-based studies from other countries supported these findings. The incidence of COVID-19 was found most commonly among adult males (median age between 34 and 59 years) (3, 24, 25). Furthermore, the highest proportion of severe cases is reported among adult patients ≥60 years of age, especially those suffer from one or more disorders such as cardiovascular and cerebrovascular diseases and diabetes (5, 6). Although the reason is not yet understood, some researchers speculate that SARS-CoV-2 is more likely to infect people with chronic comorbidities such as cardiovascular diseases (CVD) and cerebrovascular diseases and diabetes. Co-infections of bacteria and fungi may also contribute to severe manifestations (6). Males more commonly have CVDs, also more men are smokers, and their lifestyle is different (26).

Despite the 130% increase in the rate of daily reported critical COVID-19 cases during the study period, the mortality rate was not high in the KSA, and it decreased by 6.4% during the study period. Our estimates for the rates of critical cases of people infected with COVID-19 who need special care are considered extremely low compared to those published in the literature from other countries, where about 20% of all cases usually need to be hospitalized (3). In fact, this could be due to several reasons such as Saudi Arabian demographics, as the Saudi population is younger compared to European and Asian countries (27). This was also observed in other Middle Eastern countries, such as Qatar and the United Arab Emirates, where the mortality rates were low (28). In addition, the Saudi government implemented strict rules in the fight against COVID-19 including travel restrictions and lockdown of cities (29), and these measures may have helped the country to contain the spread of the virus and helped in the process of providing early recognition and treatment of cases, and therefore better outcomes (30).

This study found that the number of new COVID-19 cases decreased sharply during the period of complete lockdown (between 22 and 29 May), and started to increase again after ending the lockdown, reaching its peak on 6 June with 3,121 newly confirmed COVID-19 cases. The Saudi government eased some of the strict rules of lockdown and quarantine for the period between 22 and 29 May in order to re-open the country and minimize the socioeconomic effects of COVID19 (31), and this may have led to the pattern observed at the end of study period. In addition, this can be seen in the number of incident cases by city (Figure 2), where Riyadh and Jeddah had a doubling in the number of cases from the end of May until the end of the study period, while Mecca city, which remains under lockdown, had a more stable curve throughout the same period. However, it is important to highlight that this study is ecological and therefore, it is difficult to conclude any association or causality.

This study outlined that the epidemiological status in the KSA is getting better, which can be seen from the stable rate of active cases, specifically after 2 months from the beginning of the pandemic (12 March 2020) onwards (32). This reflects implementation of successful healthcare practices and treatment protocols. In addition, the application of international travel restrictions and household quarantine helped to slow down the spread of COVID-19 in the KSA.

This study examined the trend of COVID-19 in the KSA in terms of recovery rates, mortality rates and rates of critical cases. In addition, we presented trends of COVID-19 incidence stratified by age and gender. However, this study has some limitations. Despite the fact that this study was a population-level study at the national level, it was ecological and therefore we were unable to access data on patient level to identify other risk factors such as the presence of comorbidities, or other factors associated with COVID-19 infection. The age and gender distribution of the death cases with COVID-19 was not available in our study as this type of data was not mentioned in the Saudi Ministry of Health reports.

In conclusion, the results of this study showed that males and adults accounted for the majority of COVID-19 cases in the KSA. Moreover, our study suggests that the epidemiological status in Saudi Arabia is getting better, specifically while applying restrictive measures. Further studies are recommended to be conducted at the patient level to identify other patient groups who are at higher risk of getting infected with COVID-19, and for whom the best pharmacological intervention could be provided.

## Data Availability Statement

The original contributions presented in the study are included in the article/supplementary material, further inquiries can be directed to the corresponding author/s.

## Ethics Statement

The study was based on publicly available data and did not involve any new studies of human or animal subjects performed by any of the authors. Ethical approval was obtained for this study from the Research Ethics Committee at Najran University, Kingdom of Saudi Arabia (ref. No: 10 – 05 - 03 – 2020 EC).

## Author Contributions

MA, AN, and HAly: conceptualization, methodology, validation, writing–review and editing, and funding acquisition. AN and HAlw: software, formal analysis, data curation, writing–original draft preparation, visualization. MA, AN, MO, HAlw, and HAly investigation. MA, AN, and HAly: resources and supervision. MA, AN, HAlw and HAly: project administration. All authors agreed to be accountable for the content of the work.

## Conflict of Interest

The authors declare that the research was conducted in the absence of any commercial or financial relationships that could be construed as a potential conflict of interest.
